# Impact of a Moderate CYP3A4 Inducer (Bosentan) on Lurbinectedin Pharmacokinetics and Safety in Patients with Advanced Solid Tumors: An Open-Label, Two-Way, Crossover, Phase Ib Drug–Drug Interaction Study

**DOI:** 10.3390/ph17020182

**Published:** 2024-01-30

**Authors:** Irene Moreno, Tatiana Hernández, Emiliano Calvo, Salvador Fudio, Carmen Kahatt, Cristian Fernández, Jorge Luis Iglesias, Gema Corral, Laura Pérez-Ramos, Lola Montilla, Ali Zeaiter, Rubin Lubomirov

**Affiliations:** 1 START Madrid—CIOCC, Centro Integral Oncológico Clara Campal, Hospital Universitario HM Sanchinarro, 28050 Madrid, Spain; irene.moreno@startmadrid.com (I.M.); emiliano.calvo@startmadrid.com (E.C.); 2 START Madrid—FJD, Hospital Universitario Fundación Jiménez Díaz, 28040 Madrid, Spain; tatiana.hernandez@start-barcelona.com; 3 PharmaMar S.A., Colmenar Viejo, 28770 Madrid, Spaingcorral@pharmamar.com (G.C.);

**Keywords:** drug–drug interaction, CYP3A4, bosentan, pharmacokinetics, lurbinectedin, cancer patients, advanced solid tumors

## Abstract

This open-label, two-way, crossover, phase Ib drug–drug interaction study investigated whether the pharmacokinetics (PKs) and safety profile of lurbinectedin (LRB) are affected by co-administration of a moderate CYP3A4 inducer (bosentan, BOS) in adult patients with advanced solid tumors. Eleven patients were randomly assigned to Sequence 1 (LRB + BOS in Cycle 1 [C1] and LRB alone in Cycle 2 [C2]) or Sequence 2 (LRB alone in C1 and LRB + BOS in C2), and finally, eight patients (four per sequence) were considered evaluable for PK assessment. LRB (3.2 mg/m^2^, 1 h [h], intravenous) was administered alone or combined with multiple BOS administration (125 mg/12 h oral; 5.5 days). Co-administration with BOS decreased the systemic total exposure (area under the curve, AUC) of LRB by 21% for AUC_0–t_ and 20% for AUC_0–∞_ and increased clearance by 25%. Co-administration with BOS did not significantly modify the unbound plasma LRB PK parameters. BOS increased the conversion of LRB to its metabolite M1, with no changes on its metabolite M4. The LRB safety profile was consistent with the toxicities previously described for this drug. No differences in terms of toxicity were found between LRB with and without BOS. In summary, the magnitude of the observed changes precludes a clinically relevant effect of BOS co-administration on LRB exposure and its safety profile.

## 1. Introduction

Lurbinectedin (Zepzelca^TM^) is a novel tetrahydroisoquinoline that inhibits oncogenic transcription. It recognizes specific sequences in the DNA minor groove, where it forms adducts that ultimately lead to the generation of double-strand breaks (DSBs) [1,2,3]. Additionally, it induces the specific degradation of transcribing RNA Pol II and the eviction of transcription factors from the promoters of actively transactivated genes [4]. The generation of DSBs triggers an extended delay in transition through the S phase of the cell cycle with an arrest at the GS/M transition, ultimately leading to tumor cell death by apoptosis [5]. Lurbinectedin received U.S. Food and Drug Administration (FDA) accelerated approval in the United States in June 2020 [6,7] and later in several other countries for the treatment of adult patients with metastatic small-cell lung cancer (SCLC) with disease progression during or after platinum-based chemotherapy, based on the results of a Basket phase II study on 105 patients with previously treated SCLC [8]. 

Both the National Comprehensive Cancer Network (NCCN) guidelines [9] and the European Society for Medical Oncology (ESMO) clinical practice guidelines for SCLC [10] recommend the administration of lurbinectedin for patients with disease relapse after prior systemic platinum-based therapy. More recently, the results of other cohorts of this Basket trial have shown that lurbinectedin has relevant antitumor activity in relapsed Ewing sarcoma [11] and interesting activity in endometrial cancer, germline BRCA1/2 metastatic breast cancer, and neuroendocrine tumors [12,13,14]. 

An ongoing randomized phase III confirmatory study (LAGOON; NCT05153239) is currently evaluating lurbinectedin alone or combined with irinotecan *versus* standard-of-care therapy in second-line SCLC [15].

Cytochrome P450 3A (CYP3A) enzymes are often significant contributors to the clearance (CL) of drugs. Lurbinectedin, a structural analog of trabectedin, is primarily metabolized in the liver by CYP3A4 [16,17] and, therefore, a moderate CYP3A4 inductor such as bosentan is expected to produce the sustained induction of CYP3A4 activity over the entire lurbinectedin pharmacokinetic (PK) profile, thereby affecting clearance rates and plasma exposure. Hence, this drug–drug interaction (DDI) study assessed the exposure of 3.2 mg/m^2^ lurbinectedin given as a 1 h (h) intravenous (IV) infusion when co-administered with 125 mg bosentan twice daily (BID) in cancer patients over five and a half days. This is the first study to assess DDI using bosentan in these patients. In the previous Basket study, lurbinectedin as a single agent at a dose of 3.2 mg/m^2^ administered over 1 h without concomitant CYP3A4 inhibitors produced measurable plasma lurbinectedin concentrations for at least 160 h after the start of the infusion. Lurbinectedin at a dose of 3.2 mg/m^2^ co-administered with bosentan was expected to produce measurable concentrations in samples collected for at least the first 80 h after infusion.

In this manuscript, the results of the PK and safety profiles of lurbinectedin co-administered with bosentan are presented in comparison with lurbinectedin alone in patients with advanced solid tumors.

## 2. Results

### 2.1. Patient Disposition and Baseline Characteristics

**Figure 1:**
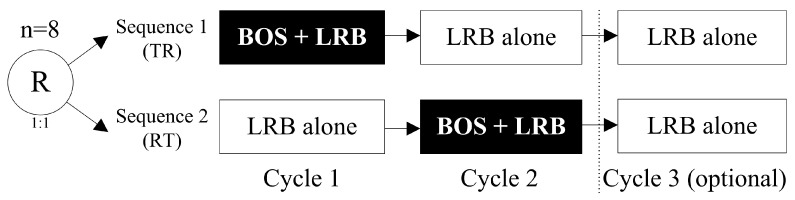
Study design. Note: Eight evaluable patients were planned to be enrolled in the study. Finally, eleven patients were included and treated: seven in Sequence 1 [TR] and four in Sequence 2 [RT]. BOS, bosentan; LRB, lurbinectedin; R; randomized; RT, Reference–Test; TR, Test–Reference.

**Figure 2:**
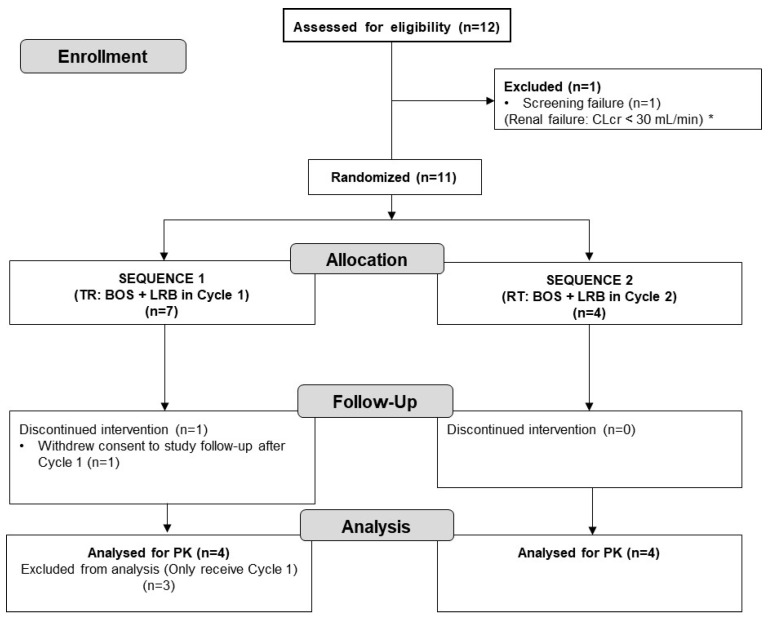
CONSORT flow diagram for the trial. * Calculated according to the Cockcroft and Gault’s formula. BOS, bosentan; CLcr, creatinine clearance, LRB, lurbinectedin; RT, Reference–Test (BOS + LRB in Cycle 2); TR, Test–Reference (BOS + LRB in Cycle 1). PK, pharmacokinetics.

Eleven patients were included and treated at two centers in Spain: seven patients were randomly assigned to Sequence 1 (**TR**: **T**est [bosentan + lurbinectedin in Cycle 1]–**R**eference [lurbinectedin alone]) and four patients to Sequence 2 (**RT**: **R**eference [lurbinectedin alone]–**T**est [bosentan + lurbinectedin in Cycle 2]). Both sequences were followed by an optional Cycle 3 of lurbinectedin alone (Figure 1). The characteristics of eligible patients are described in Section 4.2. Study Population. Six of the eleven patients (55%) discontinued treatment due to progressive disease (three patients in each sequence). In Sequence 1, one patient (14%) refused to continue treatment after Cycle 1 and withdrew consent to follow-up (Figure 2). 

Demographics and baseline characteristics by sequence are listed in Table 1. Six of the eleven included patients (55%) were female. The median age was 63 years (range, 35–74 years). The most common primary tumors were lung (n = 3; 27%) and ovarian (n = 2; 18%). The median number of disease sites at baseline was 3 (range, 2–7), with 64% of patients having ≥3 sites. The lymph nodes (n = 8; 73%), liver, and lung (n = 7; 64% each) were the most common disease sites. The median time from disease diagnosis to first infusion was 47.3 months (range, 6.8–91.6 months). Table 1 also shows the characteristics of the four patients of Sequence 1 included in the PK assessment.

### 2.2. Pharmacokinetics

A total of eight patients (four in each sequence) completed lurbinectedin PK sampling in Cycles 1 and 2 and had sufficient and interpretable PK assessments to be included in the PK analysis set.

Three treated patients in Sequence 1 were not evaluable for PK assessments because they did not receive Cycle 2. 

#### 2.2.1. Total Plasma Lurbinectedin Pharmacokinetics

**Figure 3:**
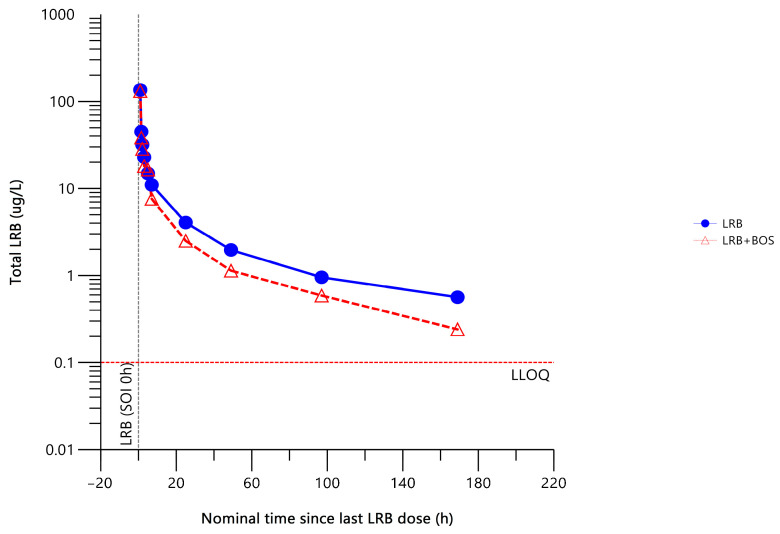
Mean total plasma concentration–time profile of lurbinectedin with (n = 8) or without (n = 8) bosentan. BOS, bosentan; h, hour; LLOQ, lower limit of quantitation; LRB, lurbinectedin; SOI, start of infusion.

The mean total plasma concentration–time profile of lurbinectedin was lower when co-administered with bosentan (Figure 3). 

Compared to lurbinectedin alone, co-administration with bosentan decreased the total plasma lurbinectedin area under the concentration–time curve (AUC) from time 0 to the time of the last quantifiable concentration (AUC_0–t_) by 21% and from time 0 to infinity (AUC_0–∞_) by 20% and increased total plasma lurbinectedin CL by 25%. These changes in lurbinectedin CL and systemic exposure were statistically significant at the 90% confidence interval (CI) level (Table 2).

#### 2.2.2. Unbound Plasma Lurbinectedin Pharmacokinetics

**Figure 4:**
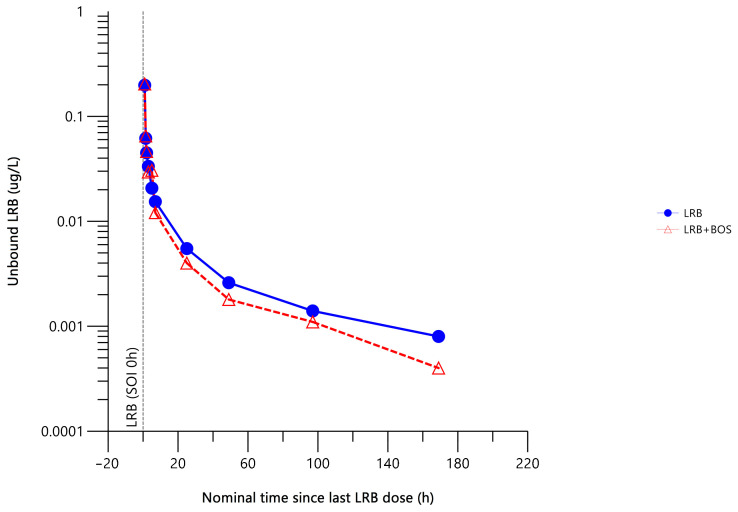
Mean unbound plasma concentration–time profile of lurbinectedin with (n = 6) or without (n = 6) bosentan. Two patients were excluded from this analysis due to extremely high unbound fraction values. BOS, bosentan; h, hour; LLOQ, lower limit of quantitation; LRB, lurbinectedin; SOI, start of infusion.

The mean unbound plasma concentration–time profiles of lurbinectedin administered alone and with bosentan are presented in Figure 4.

Co-administration with bosentan did not result in any statistically significant changes in the unbound plasma lurbinectedin PK parameters (Table 3).

#### 2.2.3. Lurbinectedin Metabolites (M1 and M4) Plasma Pharmacokinetics

Co-administration with multiple oral doses of bosentan increased the conversion of lurbinectedin to metabolite M1 (1′,3′–dihydroxy–lurbinectedin) by approximately 1.9-fold for maximum plasma concentration (C_max_) and by 2.45-fold for AUC_0–t_ compared to lurbinectedin alone at 3.2 mg/m^2^ given as a 1 h IV infusion. No changes were observed in the PK parameters of metabolite M4 (N–desmethyl–lurbinectedin, PM030047) (Table 4).

### 2.3. Safety

Safety was evaluated in the 11 treated patients. All 11 patients received bosentan plus lurbinectedin in Cycles 1 or 2, and 8 patients received lurbinectedin alone in Cycles 1, 2, or 3. A total of 23 cycles were administered (14 in Sequence 1 [TR: bosentan + lurbinectedin in Cycle 1], and 9 were in Sequence 2 [RT: bosentan + lurbinectedin in Cycle 2]), with a median number of 2 cycles (range, 1–3 cycles) per patient and a median relative dose intensity of 99.9% (range, 89.9–100.2%) in all treated patients.

All treatment-emergent adverse events (TEAEs) of any grade, regardless of relationship, are provided in Table 5. 

Table 6 shows the treatment-related adverse events (AEs) (>10% of patients or grade ≥3) and laboratory abnormalities (regardless of relationship) according to the worst grade per treatment. 

Differences in toxicities observed when lurbinectedin was administered with or without bosentan were not relevant (Table 6), considering the small number of patients treated, the different order of treatment given depending on assigned sequence (see Figure 1), and the optional Cycle 3 of lurbinectedin alone in the event of clinical benefit after the first two cycles.

No patients discontinued the study treatment due to a treatment-related AE. One patient required a lurbinectedin dose reduction to 2.6 mg/m^2^ in Cycle 3 due to grade 4 neutropenia during Cycle 2, after being treated with lurbinectedin alone (the event had an unknown relationship according to the investigator). No treatment-related deaths occurred. 

Three patients (43%) in Sequence 1 and one patient (25%) in Sequence 2 continued treatment with lurbinectedin alone under compassionate use after the completion of the optional Cycle 3.

## 3. Discussion

This open-label, two-way, crossover, phase Ib DDI study compared the PK and safety profile of lurbinectedin alone and co-administered with the moderate CYP3A4 inducer bosentan in patients with advanced solid tumors. 

Bosentan is an orally active non-peptide pyrimidine derivative that competitively antagonizes the binding of endothelin–1 (ET–1) to both ET_A_ and ET_B_ receptor subtypes and irreversibly blocks their activities [18]. It is mainly metabolized in the liver by the cytochromes P450 enzymes CYP2C9 and CYP3A4, and the hepatic metabolism followed by biliary metabolites excretion represents the major pathway of elimination in humans [19]. Lurbinectedin is primarily metabolized by the CYP3A4 isoenzyme; therefore, the potential effects of bosentan on the PK profile of lurbinectedin at a dose of 3.2 mg/m^2^ as a 1 h IV infusion, if given concomitantly with multiple oral doses of bosentan, have been assessed in this trial. Of note, this is the first DDI study using this moderate CYP3A4 inducer in cancer patients. A crossover design was used in this study to reduce treatment bias and allow for intra-subject comparisons and control.

Compared with lurbinectedin alone, co-administration with bosentan marginally reduced total plasma lurbinectedin systemic exposure by 20% (90% CI: 2–35%) without changing unbound plasma lurbinectedin systemic exposure. Total plasma lurbinectedin clearance increased by 25% (90% CI: 2–54%), mostly by increasing its conversion to metabolite M1. Metabolites M1 and M4 are the most relevant circulating lurbinectedin metabolites in the blood [20], and they were the only ones assessed in this DDI study. Overall, the magnitude of the observed changes suggests that the co-administration of bosentan has no clinically relevant effects on lurbinectedin exposure in patients with advanced solid tumors.

The study design of this trial was adequate for characterizing the PK profile of total and unbound lurbinectedin administered alone and in combination with bosentan. The results suggest that lurbinectedin dosage adjustments are not necessary when a moderate CYP3A4 inducer is co-administered. 

The safety profile observed herein for lurbinectedin alone at 3.2 mg/m^2^ as a 1 h IV infusion every three weeks (q3wk) was consistent with that reported for this same dose and schedule in previous clinical trials in patients with advanced cancer, with the most common treatment-related AEs being neutropenia, nausea, vomiting, and fatigue [21]. Lurbinectedin with and without bosentan was well-tolerated in the present study. The safety profile of lurbinectedin when co-administered with bosentan was similar to that of lurbinectedin administered alone.

In summary, co-administration with a moderate CYP3A4 inducer (bosentan) decreased the systemic total exposure of lurbinectedin by 21% for AUC_0–t_ and 20% for AUC_0–∞_ and increased clearance by 25%. No statistically significant modifications were observed in the unbound plasma lurbinectedin PK parameters. Furthermore, no toxicity differences were found between lurbinectedin alone and lurbinectedin with bosentan. This trial was the first DDI study with bosentan used as a moderate CYP3A4 inducer in cancer patients. The findings from this study show that dose modifications are not required when patients with advanced or metastatic solid tumors need to take lurbinectedin concomitantly with moderate CYP3A inducers.

## 4. Materials and Methods

### 4.1. Study Design and Settings

This study was designed as an open-label, two-way, crossover, phase Ib DDI study of lurbinectedin and bosentan in adult patients with advanced solid tumors. The study included: (i) a screening phase (within 14 days prior to any study procedure); (ii) a treatment phase consisting of two lurbinectedin cycles (one with and one without bosentan co-administration, in a different order, depending on the assigned sequence of treatment) and an optional third cycle of lurbinectedin alone for patients who met the criteria for treatment continuation and showed clinical benefit after the first two cycles; and (iii) a follow-up phase after the last dose of lurbinectedin. 

Patients were randomized to receive either Sequence 1 (TR: Test–Reference) of bosentan (125 mg tablets; BID for five consecutive days and once daily on Day 1 [i.e., the day of lurbinectedin administration]) co-administered with lurbinectedin (3.2 mg/m^2^, 1 h IV infusion q3wk) (Cycle 1), followed by two consecutive cycles (Cycle 2 and the optional Cycle 3) of lurbinectedin alone (3.2 mg/m^2^, 1 h, IV infusion q3wk), or Sequence 2 (RT: Reference–Test) of lurbinectedin alone (Cycle 1), followed by bosentan and lurbinectedin (Cycle 2) and lurbinectedin alone (optional Cycle 3) (see above Figure 1). Based on exposure and the safety data of the first three treated patients who completed Cycles 1 and 2, the dose of lurbinectedin was 3.2 mg/m^2^ for all patients when administered with and without bosentan. This dose has been used in studies of single-agent lurbinectedin conducted in the US and Europe with cancer patients [8,22,23], especially in SCLC, and is the approved dose of lurbinectedin for relapsed SCLC patients with disease progression during or after platinum-based chemotherapy.

In the co-administration cycles, bosentan was taken orally in the morning and the evening (after breakfast and dinner, respectively) for five consecutive days, self-administered at home starting from Day − 5 (i.e., five days before lurbinectedin infusion) to Day − 1 (i.e., the day before lurbinectedin infusion), following prescribing information recommendations, and given at the study center on Day 1 (day of lurbinectedin infusion). On Day 1, bosentan was given immediately prior to the start of the lurbinectedin infusion. In fact, bosentan had to be administered after collecting the first bosentan PK sample and before the start of lurbinectedin infusion (−15 min [min] to −1 min). In case of lurbinectedin delay (≤2 days), bosentan could be administered for a maximum of 7 and a half days (Supplementary Figure S1). In Sequence 1 (TR), bosentan for self-administration and the patient’s diary were given to the patient on Day −6, with a −2/+1 daytime window (i.e., from Day −8 to Day −5). In Sequence 2 (RT), bosentan and the patient’s diary were given to the patient on Day –6 of Cycle 2, with a –2/+1 daytime window (i.e., from Day 14 of Cycle 1 to Day −5 of Cycle 2) (Figure S1). Follow-up calls by phone (or other methods) were conducted by the study center to remind or confirm that the patients had taken the morning and evening doses of bosentan when they were not at the study center. Compliance with bosentan administration was confirmed using a patient’s diary.

The study was conducted according to the Helsinki Declaration Guidelines and approved by the Spanish Agency of Medicines and Medical Devices (AEMPS) (protocol code: 2020-002595-12 approved on 6 November 2020) and the Independent Ethics Committee of HM Hospitals (protocol code 20.07.1663-GHM approved on 2 September 2020). It was registered with the EU Clinical Trials Register EudraCT (2020-002595-12) and ClinicalTrials.gov Trials Register (NCT05072106). The study followed the ICH Good Clinical Practice (GCP) guidelines and applicable regulatory requirements and was conducted in compliance with the study protocol.

### 4.2. Study Population

Patients with advanced solid tumors were included and treated between January and October 2021. Eligible patients were men and women aged ≥18 years with pathologically confirmed advanced solid malignancies, who had recovered from previous toxicities to grade ≤1 (excluding alopecia and grade 1/2 asthenia or fatigue) and had a life expectancy longer than 3 months, with an Eastern Cooperative Oncology Group (ECOG) performance status score of ≤1 and adequate organ function. Women were postmenopausal, surgically sterile, abstinent, or practicing a highly effective method of birth control (including breastfeeding) throughout the study and for six months thereafter. Men used an adequate contraception method (e.g., vasectomy, double barrier, partner using effective contraception) during treatment and for four months after treatment.

Major exclusion criteria included the prior use of strong or moderate inhibitors or inducers of CYP3A4 activity within three weeks prior to Day 1 of Cycle 1 and the use of CYP3A4 substrates for which concomitant administration with moderate CYP3A4 inductor was contraindicated. Patients with CNS metastasis, cirrhosis, alcohol-induced steatosis, chronic active hepatitis infection, significant cardiovascular conditions and medical conditions such as obstructive cholestatic liver disease (suitable for stenting procedure) or biliary sepsis in the past two months, active COVID-19 disease, or a psychiatric disorder were also excluded.

### 4.3. Randomization 

This is an open-label study; therefore, the blinding of treatment was not performed.

Eligible patients were randomized after the 14-day baseline period. Block randomization was used to reduce bias in the assignment of patients to treatment sequence groups and to achieve balance in the allocation of patients across treatment sequence groups, thereby enhancing the validity of statistical comparisons across the treatment sequence groups. Randomization was implemented with Medidata Rave Randomization and Trial Supply Management (RTSM). A randomization list was generated to randomly allocate patients to Sequence 1 (TR: Test–Reference; bosentan + lurbinectedin in Cycle 1) or Sequence 2 (RT: Reference–Test; bosentan + lurbinectedin in Cycle 2). The AEs were evaluated before treatment allocation was revealed.

### 4.4. Pharmacokinetic Evaluations

#### 4.4.1. Sample Collection

Blood samples for the PK analysis of lurbinectedin and its metabolites (i.e., 1’,3’–dihydroxy-lurbinectedin [M1] and N-desmethyl-lurbinectedin [M4, PM030047]) were collected in Cycle 1 and Cycle 2 (Sequence 2 [RT] and Sequence 1 [TR], respectively), with a schedule of 11 samples for lurbinectedin and the first 8 samples for their metabolites: at pre-dose, 5 min prior to the end of the lurbinectedin infusion, and at 0.5 h, 1 h, 2 h, 4 h, 6 h, 24 h, 48 h, 96 h and 168 h post-dose on Days 1, 2, 3, 5 and 8. Blood samples for the PK analysis of bosentan were collected in cycles with bosentan co-administration (i.e., Cycle 1 of Sequence 1 [TR] and Cycle 2 of Sequence 2 [RT]), with a schedule of four samples: at pre-dose and at 1 h, 4 h, and 24 h post-bosentan dosing on Days 1 and 2.

#### 4.4.2. Pharmacokinetic Parameters

The effect of bosentan co-administration on the PK profile of lurbinectedin compared with that of lurbinectedin alone was assessed using the C_max_ and the AUC_0–∞_ of total plasma lurbinectedin as primary study PK endpoints. Secondary PK endpoints included AUC_0–t_, CL, terminal elimination half-life (t_1/2_) and the volume of distribution at steady state (V_ss_). Unbound AUC_u,0–∞_, AUC_u,0–t_, C_u,max_, CL_u_, V_ss,u_ and t_1/2,u_ were also estimated. Plasma PK parameters were calculated using a standard non-compartmental analysis approach using Phoenix^®^ WinNonlin^®^ v6.4 (Certara USA, Inc., Princeton, NJ, USA).

#### 4.4.3. Bioanalytical Procedures

Total plasma concentrations of lurbinectedin were determined using a validated Ultra Performance Liquid Chromatography coupled to tandem Mass Spectrometry (UPLC-MS/MS) method (Dynakin, S.L., Spain) [24]. Lurbinectedin unbound fraction (LRB fu) in plasma was determined by rapid equilibrium dialysis (RED) and quantification using a validated UPLC-MS/MS method. Plasma concentrations of lurbinectedin metabolites M1 and M4 were quantified using a validated UPLC-MS/MS method. The lower limit of quantification of total lurbinectedin, unbound lurbinectedin, and M1 and M4 metabolites were 0.1 ng/mL, 0.02%, 0.5 and 0.1 ng/mL, respectively. Bosentan total plasma concentrations were determined using a Liquid Chromatography coupled to tandem Mass Spectrometry (LC-MS/MS) method (Anapharm Europe, S.L.U., Barcelona, Spain). The lower limit of quantification for bosentan was 5.0 ng/mL.

### 4.5. Safety Evaluations 

Patients were evaluable for safety if they had received at least one partial or complete infusion of lurbinectedin. AEs were graded as per the National Cancer Institute-Common Terminology Criteria of Adverse Events (NCI-CTCAE) v.5.0 and coded using the Medical Dictionary for Regulatory Activities (MedDRA) v.23.0. Safety evaluations included assessments of AEs, deaths, clinical laboratory tests, vital signs measurements and physical examinations. TEAEs were defined as any AE aggravated in severity from baseline or having their onset between the first dose of the study drug and 31 days (±10 days) after the last treatment dose; death or date of further therapy were also considered TEAEs. 

### 4.6. Statistical Methods

#### 4.6.1. Sample Size

This study was designed to assess the potential effects of bosentan on the PK profile of lurbinectedin in patients with advanced malignancies. Based on feasibility and clinical considerations, at least eight patients were expected to complete all study procedures, which would be sufficient to estimate the DDI of bosentan with lurbinectedin. The intra-subject coefficient of variation (CV) for the PK parameters of lurbinectedin was estimated to be more than 30%. The half-width of the 90% CI for [(Test: bosentan plus lurbinectedin)/(Reference: lurbinectedin alone)] comparison on the log-scale was extended by 0.389 from the observed differences in means, assuming that the intra-subject CV was around 40%. The 90% CI was used to help with the interpretation of the results. This half-width corresponds to a 90% CI in the range of 70% and 147%, assuming the ratio of the means is equal to unity for each PK parameter. 

#### 4.6.2. Statistical Analysis

Descriptive statistics were used to summarize the patients’ baseline characteristics/variables.

Only patients who completed the study with sufficient and interpretable PK parameters to calculate the non-compartmental PK parameters were included in the statistical comparison of plasma exposure to lurbinectedin.

The primary parameter of interest for the statistical analysis was the plasma dose-normalized AUC (_0–∞_) of lurbinectedin (AUC_(0–t)_ was used instead if AUC (_0–∞_) could not be calculated due to insufficient available data). The analysis compared the log-transformed AUC for lurbinectedin in combination with bosentan (Test) *versus* lurbinectedin alone (Reference). A mixed-effects model was fit to the data with log-transformed AUC as the dependent variable; treatment (Test or Reference), period (Cycle 1 or Cycle 2) and sequence (Sequence 1 or Sequence 2) as fixed effects; and patient (nested in the sequence) as a random effect. The estimated least-square means and intra-subject variability from the mixed-effects model were used to construct 90% CIs for the difference in means on the log scale between treatments (Test or Reference). The adjusted mean differences and the 90% CIs were exponentiated to obtain estimates of the ratio of adjusted geometric means (Test/Reference) and 90% CIs for the ratios. A large difference (e.g., a two-fold difference in CIs and least-square means) was considered to be suggestive of a clinically relevant effect of bosentan co-administration on lurbinectedin exposure. Similar models were fitted to the data with a dose-normalized AUC_(0–t)_ and C_max_, and in CL, the V_ss_ and t_1/2_ of lurbinectedin or metabolites-to-parent exposure PK parameters ratio were used as the dependent variable. For plasma protein binding, a similar model was fitted to the data with the dose-normalized AUC_u_ as the dependent variable. Safety variables were summarized descriptively. Safety analyses were presented by the treatment group (Test: bosentan in combination with lurbinectedin *versus* Reference: lurbinectedin alone). The differences in dose-normalized natural log-transformed PK parameters for bosentan with lurbinectedin *versus* lurbinectedin alone, presented as geometric means (geometric percent coefficient variation, CV%), were assessed using an analysis of variance (ANOVA) model with treatment (T: Test or R: Reference), period (Cycle 1 or 2) and sequence (Sequence 1 [TR] or 2 [RT]) as fixed effects and patient (sequence) as a random effect. The least-squares geometric mean ratio was calculated by dividing the geometric mean of bosentan by the geometric mean of lurbinectedin. All statistical analyses were performed using SAS^®^ v.9.4 (SAS Institute Inc., Cary, NC, USA). 

## Figures and Tables

**Table 1:** Demographics and baseline characteristics by sequence.

	Sequences			Total
	Seq. 1 (TR: BOS + LRB Cycle 1)		Seq. 2 (RT: BOS + LRB Cycle 2)	
	(n = 7)	(n = 4) ^a^	(n = 4)	(N = 11)
**Gender**				
Male	3 (43%)	2 (50%)	2 (50%)	5 (45%)
Female	4 (57%)	2 (50%)	2 (50%)	6 (55%)
**Median age, years (range)**	67 (58–74)	66 (61–69)	60 (35–65)	63 (35–74)
**ECOG performance status**				
0	4 (57%)	3 (75%)	2 (50%)	6 (55%)
1	3 (43%)	1 (25%)	2 (50%)	5 (45%)
**Median BSA, m^2^ (range)**	1.7 (1.5–2.0)	1.9 (1.5–2.0)	1.9 (1.7–2.2)	1.7 (1.5–2.2)
**Stage at diagnosis**				
Early	4 (57%)	2 (50%)	1 (25%)	5 (45%)
Locally advanced	2 (29%)	2 (50%)	1 (25%)	3 (27%)
Metastatic	1 (14%)	–	2 (50%)	3 (27%)
**Primary tumors**				
Lung	1 (14%)	1 (25%)	2 (50%)	3 (27%) ^b^
Ovarian	1 (14%)	–	1 (25%)	2 (18%)
Breast	1 (14%)	1 (25%)	–	1 (9%)
Cervical	1 (14%)	1 (25%)	–	1 (9%)
Colon	1 (14%)	1 (25%)	–	1 (9%)
Gall bladder	1 (14%)	–	–	1 (9%)
Mesothelioma	1 (14%)	–	–	1 (9%)
Neuroendocrine tumor	–	–	1 (25%)	1 (9%)
**Number of sites of disease involvement**				
Median (range)	3 (2–4)	2 (2–4)	6 (2–7)	3 (2–7)
**Sites of disease**				
Lymph node	4 (57%)	2 (50%)	4 (100%)	8 (73%)
Liver	4 (57%)	3 (75%)	3 (75%)	7 (64%)
Lung	4 (57%)	2 (50%)	3 (75%)	7 (64%)
Peritoneum	4 (57%)	2 (50%)	1 (25%)	5 (45%)
Bone	1 (14%)	1 (25%)	3 (75%)	4 (36%)
CNS	–	–	3 (75%)	3 (27%)
Adrenal	–	–	2 (50%)	2 (18%)
Pleura	1 (14%)	–	1 (25%)	2 (18%)
Soft tissue	1 (14%)	–	–	1 (9%)
Pancreas	–	–	1 (25%)	1 (9%)
**Time from diagnosis to first infusion (months)**				
Median (range)	47.7 (30.5–91.6)	41.9 (30.5–91.6)	13.7 (6.8–59.4)	47.3 (6.8–91.6)
**Prior treatment for advanced disease (prior chemotherapy lines)**				
Median (range)	3 (3–5)	3 (3–4)	2.5 (1–3)	3 (1–5)

Data shown are n (%) of treated patients by sequence (Sequence 1 [TR] versus Sequence 2 [RT]), except for median (range). Sequence 1 (TR): Bosentan (125 mg/12 h oral; 5.5 days) + lurbinectedin (C1) followed by C2 and C3 of lurbinectedin alone; Sequence 2 (RT): lurbinectedin alone (C1) followed by bosentan and lurbinectedin co-administration (C2) and lurbinectedin alone (C3). Lurbinectedin was administered at 3.2 mg/m² as 1 h IV infusion q3wk for all patients when given with and without bosentan. ^a^ Patients from Sequence 1 included in the PK assessment. ^b^ SCLC (n = 2 patients) and NSCLC (n = 1 patient). BOS, bosentan; BSA, body surface area; C, cycle; CNS, central nervous system; ECOG, Eastern Cooperative Oncology Group; h, hour; IV, intravenous; LRB, lurbinectedin; NSCLC, non-small-cell lung cancer; PS, performance status; q3wk, every three weeks; RT, Reference–Test (BOS + LRB in C2); Seq.1, Sequence 1; Seq. 2, Sequence 2; SCLC, small-cell lung cancer; TR, Test–Reference (BOS + LRB in C1).

**Table 2:** Total plasma pharmacokinetic parameters of lurbinectedin alone and co-administered with bosentan.

PK Parameter (Units)	Treatment ^b^	Geometric Mean (CV%)	Ratio (%) ^c^	90% CI (%) ^d^	Intra-Subject CV (%)
**C_max_ (µg/L/mg) ^a^**	**BOS + LRB (T)**	20.71 (54.81)	96.83	(81.09–115.62) *	18.41
	**LRB (R)**	21.39 (49.56)			
**AUC_0–t_ (µg·h/L/mg) ^a^**	**BOS + LRB (T)**	56.33 (76.47)	79.20	(64.12–97.82) *	21.99
	**LRB (R)**	71.13 (84.97)			
**AUC0-∞ (µg·h/L/mg) ^a^**	**BOS + LRB (T)**	58.83 (75.83)	79.81	(64.78–98.32) *	21.72
	**LRB (R)**	73.71 (86.93)			
**CL (L/h)**	**BOS + LRB (T)**	17.00 (75.83)	125.30	(101.71–154.36) *	21.72
	**LRB (R)**	13.57 (86.93)			
**t_1/2_ (h)**	**BOS + LRB (T)**	35.51 (61.54)	104.93	(69.49 –158.44)	44.40
	**LRB (R)**	33.84 (35.26)			
**V_ss_ (L)**	**BOS + LRB (T)**	412.94 (78.11)	106.79	(74.63–152.8)	38.17
	**LRB (R)**	386.69 (65.5)			

A natural log transformation for AUC and C_max_ was used prior to ANOVA. Geometric means, geometric means ratio and its 90% CI were back-transformed to the original scale. All PK parameters are presented as geometric mean (geometric percent coefficient variation, CV%). * Statistically significant at the 90% CI. ^a^ Dose-normalized PK parameter. ^b^ Test, n = 8 patients; Reference, n = 8 patients. ^c^ Ratio = least-squares geometric mean ratio. Geometric mean ratio was calculated dividing geometric mean of lurbinectedin + bosentan by geometric mean of lurbinectedin. ^d^ Based on an ANOVA with treatment (T: Test or R: Reference), period (Cycle 1 or 2) and sequence (Sequence 1 [TR] or 2 [RT]) as fixed effects and patient (sequence) as a random effect in the model. ANOVA, analysis of variance; AUC, area under the concentration–time curve; BOS, bosentan; CI, confidence interval; CL, clearance; C_max_, maximum plasma concentration; CV, coefficient of variation; LRB, lurbinectedin; n, number of patient with PK parameter included; PK, pharmacokinetic; R, Reference; RT, Reference–Test (BOS + LRB in Cycle 2); t_1/2_, terminal half-life; T, Test; TR, Test–Reference (BOS + LRB in Cycle 1); V_ss_, volume of distribution at steady state.

**Table 3:** Total unbound plasma pharmacokinetic parameters of lurbinectedin alone and co-administered with bosentan.

PK Parameter (Units)	Treatment ^b^	Geometric Mean (CV%)	Ratio (%) ^c^	90% CI (%) ^d^	Intra-Subject CV (%)
**C_max_ (µg/L/mg) ^a^**	**BOS + LRB (T)**	0.0324 (51.77)	88.44	(65.62–119.19)	23.16
	**LRB (R)**	0.0332 (19.18)			
**AUC_0–t_ (µg·h/L/mg) ^a^**	**BOS + LRB (T)**	0.0907 (72.73)	80.26	(54.26–118.72)	30.68
	**LRB (R)**	0.1099 (58.24)			
**AUC_0–∞_ (µg·h/L/mg) ^a^**	**BOS + LRB (T)**	0.095 (73.04)	81.08	(54.8–119.96)	30.70
	**LRB (R)**	0.114 (61.09)			
**CL (L/h)**	**BOS + LRB (T)**	10,530.93 (73.04)	123.34	(83.36–182.49)	30.70
	**LRB (R)**	8773.36 (61.09)			
**t_1/2_ (h)**	**BOS + LRB (T)**	37.41 (73.98)	120.50	(64.79–224.13)	50.36
	**LRB (R)**	32.33 (39.36)			
**V_ss_ (L)**	**BOS + LRB (T)**	266,905.87 (73.09)	121.90	(68.59–216.62)	46.27
	**LRB (R)**	241,282.16 (23.18)			

A natural log transformation for AUC and C_max_ was used prior to ANOVA. Geometric means, geometric means ratio and its 90% CI were back-transformed to the original scale. All PK parameters are presented as geometric mean (geometric percent coefficient variation, CV%). ^a^ Dose-normalized PK parameter. ^b^ Test, n = 6 patients; Reference, n = 6 patients. Two patients were excluded from the analysis due to extremely high unbound fraction values. ^c^ Ratio = least-squares geometric mean ratio. Geometric mean ratio was calculated dividing geometric mean of lurbinectedin + bosentan by geometric mean of lurbinectedin. ^d^ Based on an ANOVA with treatment (T: Test or R: Reference), period (Cycle 1 or 2) and sequence (Sequence 1 [TR] or 2 [RT]) as fixed effects and patient (sequence) as a random effect in the model. ANOVA, analysis of variance; AUC, area under the concentration–time curve; BOS, bosentan; CI, confidence interval; CL, clearance; C_max_, maximum plasma concentration; CV, coefficient of variation; LRB, lurbinectedin; n, number of patient with PK parameter included; PK, pharmacokinetic; R, Reference; RT, Reference–Test (BOS + LRB in Cycle 2); t_1/2_, terminal half-life; T, Test; TR, Test–Reference (BOS + LRB in Cycle 1); V_ss_, volume of distribution at steady state.

**Table 4:** Total plasma pharmacokinetic parameters of lurbinectedin metabolites (M1 and M4) with lurbinectedin alone and co-administered with bosentan.

Lurbinectedin Metabolites	MPR of PK Parameter (Units) ^a^	Treatment (n)	Geometric Mean (CV%)	Ratio (%) ^c^	90% CI (%) ^d^	Intra-Subject CV (%)
**M1** (*1′,3′-dihydroxy-lurbinectedin*)	**C_max_ (µg/L/mg)**	**BOS + LRB (T)** (n = 8)	0.3849 (92.13)	188.13	(132.93–266.26)	33.84
		**LRB (R)** (n = 7) ^b^	0.268 (78.43)			
	**AUC_0–t_ (µg·h/L/mg)**	**BOS + LRB (T)** (n = 8)	0.728 (236.22)	245.46	(133.40–451.65)	63.43
		**LRB (R)** (n = 7) ^b^	0.6916 (114.55)			
**M4** (*PM030047, N-desmethyl-lurbinectedin*)	**C_max_ (µg/L/mg)**	**BOS + LRB (T)** (n = 8)	0.5617 (103.28)	104.94	(94.07–117.05)	11.28
		**LRB (R)** (n = 8)	0.5528 (101.15)			
	**AUC_0–t_ (µg·h/L/mg)**	**BOS + LRB (T)** (n = 8)	0.9962 (267.9)	91.02	(71.39–116.04)	25.39
		**LRB (R)** (n = 8)	1.382 (390.96)			

A natural log transformation for AUC and C_max_ was used prior to ANOVA. Geometric means, geometric means ratio and its 90% CI were back-transformed to the original scale. ^a^ Dose-normalized PK parameter. ^b^ Metabolite M1 plasma samples were below the limit of quantification in one patient in the LRB cycle. ^c^ Ratio = least-squares geometric mean ratio. Geometric mean ratio was calculated dividing geometric mean of lurbinectedin + bosentan by geometric mean of lurbinectedin. ^d^ Based on an ANOVA with treatment (T: Test or R: Reference), period (Cycle 1 or 2) and sequence (Sequence 1 [TR] or 2 [RT]) as fixed effects and patient (sequence) as a random effect in the model. ANOVA, analysis of variance; AUC, area under the concentration–time curve; BOS, bosentan; CI, confidence interval; C_max_, maximum plasma concentration; CV, coefficient of variation; LRB, lurbinectedin; MPR, metabolite/parent ratio; n, number of patient with PK parameter included; NA, not applicable; PK, pharmacokinetic; R, Reference; RT, Reference–Test (BOS + LRB in Cycle 2); T, Test; TR, Test–Reference (BOS + LRB in Cycle 1).

**Table 5:** Treatment-emergent adverse events (regardless of relationship), worst grade per treatment.

	BOS + LRB (n = 11)			LRB Alone (n = 8)		
NCI-CTCAE Grade	All	3	4	All	3	4
**Infections and infestations**						
Bacteremia	–	–	–	1 (12.5)	–	–
Pneumonia	1 (9.1)	–	–	–	–	–
Upper respiratory tract infection	1 (9.1)	–	–	–	–	–
**Nervous system disorders**						
Monoparesis	1 (9.1)	1 (9.1)	–	–	–	–
Paresthesia	1 (9.1)	–	–	–	–	–
**Vascular disorders**						
Venous thrombosis	–	–	–	1 (12.5)	–	–
Hot flush	1 (9.1)	–	–	–	–	–
**Respiratory, thoracic and mediastinal disorders**						
Cough	1 (9.1)	–	–	–	–	–
Dyspnea	2 (18.2)	–	–	–	–	–
**Gastrointestinal disorders**						
Diarrhea	1 (9.1)	–	–	1 (12.5)	–	–
Nausea	2 (18.2)	1 (9.1)	–	1 (12.5)	–	–
Constipation	1 (9.1)	–	–	–	–	–
Dyspepsia	1 (9.1)	–	–	–	–	–
Gastritis	1 (9.1)	–	–	–	–	–
Esophagitis	1 (9.1)	1 (9.1)	–	–	–	–
Vomiting	2 (18.2)	1 (9.1)	–	–	–	–
**Musculoskeletal and connective tissue disorders**						
Back pain	1 (9.1)	–	–	–	–	–
Bone pain	1 (9.1)	–	–	–	–	–
**General disorders and administration site conditions**						
Fatigue	5 (45.5)	1 (9.1)	–	1 (12.5)	–	–
Pain	–	–	–	1 (12.5)	–	–
Edema peripheral	1 (9.1)	–	–	–	–	–
**Investigations**						
Weight decreased	1 (9.1)	–	–	–	–	–
**Product issues**						
Thrombosis in device	1 (9.1)	1 (9.1)	–	–	–	–

Values are n (%) of patients. BOS, bosentan; LRB, lurbinectedin; NCI–CTCAE, National Cancer Institute Common Terminology Criteria for Adverse Events.

**Table 6:** Treatment-related adverse events (>10% of patients or grade ≥3) and laboratory abnormalities (regardless of relationship), worst grade per treatment.

	BOS + LRB (n = 11)			LRB Alone (n = 8)		
NCI-CTCAE Grade	All	3	4	All	3	4
**Treatment-related AEs**						
Nausea	1 (9.1)	–	–	1 (12.5)	–	–
**Hematological laboratory abnormalities (regardless of relationship)**						
Anemia	10 (90.9)	1 (9.1)	–	8 (100.0)	1 (12.5)	–
Leukopenia	5 (45.5)	–	–	6 (75.0)	4 (50.0)	–
Lymphopenia	11 (100.0)	3 (27.3)	–	8 (100.0)	3 (37.5)	–
Neutropenia	3 (27.3)	–	–	5 (62.5)	2 (25.0)	1 (12.5)
Thrombocytopenia	4 (36.4)		1 (9.1)	4 (50.0)	–	–
**Biochemical laboratory abnormalities (regardless of relationship)**						
ALT increased	4 (36.4)	–	–	2 (25.0)	–	–
AP increased	3 (27.3)	–	–	2 (25.0)	–	–
AST increased	3 (27.3)	–	–	1 (12.5)	–	–
Creatinine increased	3 (27.3)	–	–	3 (37.5)	–	–
GGT increased	2 (18.2)	1 (9.1)	–	3 (37.5)	–	–

Values are n (%) of patients. ALT, alanine aminotransferase; AP, alkaline phosphatase; AST, aspartate aminotransferase; BOS, bosentan; GGT, gamma-glutamyltransferase; LRB, lurbinectedin; NCI-CTCAE, National Cancer Institute Common Terminology Criteria for Adverse Events.

## Data Availability

The original contributions presented in the study have been included in the article and Supplementary Material; further inquiries can be directly addressed to the corresponding author.

## Supplementary Materials

The following supporting information can be downloaded at: https://www.mdpi.com/article/10.3390/ph17020182/s1, Figure S1: Schematic diagram of trial design.

## References

[1] Bueren-Calabuig J.A., Giraudon C., Galmarini C.M., Egly J.M., Gago F. (2011). Temperature-induced melting of double-stranded DNA in the absence and presence of covalently bonded antitumour drugs: Insight from molecular dynamics simulations. Nucleic Acids Res..

[2] Harlow M.L., Maloney N., Roland J., Navarro M.J.G., Easton M.K., Kitchen-Goosen S.M., Boguslawski E.A., Madaj Z.B., Johnson B.K., Bowman M.J. (2016). Lurbinectedin Inactivates the Ewing Sarcoma Oncoprotein Ews-Fli1 by Redistributing It within the Nucleus. Cancer Res..

[3] Jimeno A., Sharma M.R., Szyldergemajn S., Gore L., Geary D., Diamond J.R., Teruel C.F., Matos-Pita A.S., Iglesias J.L., Cullell-Young M. (2017). Phase I study of lurbinectedin, a synthetic tetrahydroisoquinoline that inhibits activated transcription, induces DNA single- and double-strand breaks, on a weekly × 2 every-3-week schedule. Investig. New Drugs.

[4] Santamaria Nunez G., Robles C.M., Giraudon C., Martinez-Leal J.F., Compe E., Coin F., Aviles P., Galmarini C.M., Egly J.M. (2016). Lurbinectedin Specifically Triggers the Degradation of Phosphorylated Rna Polymerase Ii and the Formation of DNA Breaks in Cancer Cells. Mol. Cancer Ther..

[5] Leal J.F., Martinez-Diez M., Garcia-Hernandez V., Moneo V., Domingo A., Bueren-Calabuig J.A., Negri A., Gago F., Guillen-Navarro M.J., Aviles P. (2010). Pm01183, a New DNA Minor Groove Covalent Binder with Potent in Vitro and in Vivo Anti-Tumour Activity. Br. J. Pharmacol..

[6] Markham A. (2020). Lurbinectedin: First Approval. Drugs.

[7] Singh S., Jaigirdar A.A., Mulkey F., Cheng J., Hamed S.S., Li Y., Liu J., Zhao H., Goheer A., Helms W.S. (2021). Fda Approval Summary: Lurbinectedin for the Treatment of Metastatic Small Cell Lung Cancer. Clin. Cancer Res..

[8] Trigo J., Subbiah V., Besse B., Moreno V., Lopez R., Sala M.A., Peters S., Ponce S., Fernandez C., Alfaro V. (2020). Lurbinectedin as Second-Line Treatment for Patients with Small-Cell Lung Cancer: A Single-Arm, Open-Label, Phase 2 Basket Trial. Lancet Oncol..

[9] Nccn Clinical Practice Guidelines in Oncology. Small Cell Lung Cancer. Version 1.2024. https://www.nccn.org/guidelines/recently-published-guidelines.

[10] Dingemans A.M., Früh M., Ardizzoni A., Besse B., Faivre-Finn C., Hendriks L.E., Lantuejoul S., Peters S., Reguart N., Rudin C.M. (2021). Small-Cell Lung Cancer: Esmo Clinical Practice Guidelines for Diagnosis, Treatment and Follow-Up. Ann. Oncol..

[11] Subbiah V., Braña I., Longhi A., Boni V., Delord J.-P., Awada A., Boudou-Rouquette P., Sarantopoulos J., Shapiro G.I., Elias A. (2022). Antitumor Activity of Lurbinectedin, a Selective Inhibitor of Oncogene Transcription, in Patients with Relapsed Ewing Sarcoma: Results of a Basket Phase II Study. Clin. Cancer Res..

[12] Kristeleit R., Leary A., Delord J.P., Moreno V., Oaknin A., Castellano D., Shappiro G.I., Fernández C., Kahatt C., Alfaro V. (2023). Lurbinectedin in patients with pretreated endometrial cancer: Results from a phase 2 basket clinical trial and exploratory translational study. Investig. New Drugs.

[13] Longo-Muñoz F., Castellano D., Alexandre J., Chawla S.P., Fernández C., Kahatt C., Alfaro V., Siguero M., Zeaiter A., Moreno V. (2022). Lurbinectedin in patients with pretreated neuroendocrine tumours: Results from a phase II basket study. Eur. J. Cancer.

[14] Boni V., Pistilli B., Brana I., Shapiro G.I., Trigo J., Moreno V., Castellano D., Fernandez C., Kahatt C., Alfaro V. (2022). Lurbinectedin, a Selective Inhibitor of Oncogenic Transcription, in Patients with Pretreated Germline Brca1/2 Metastatic Breast Cancer: Results from a Phase Ii Basket Study. ESMO Open.

[15] Besse B., Paz-Ares L.G., Peters S., Cappuzzo F., Reck M., Calles A., Califano R., Lopez-Vilariño J.A., Veramendi S., Kahatt C.M. (2023). A Phase Iii Study of Lurbinectedin Alone or in Combination with Irinotecan Vs Investigator’s Choice (Topotecan or Irinotecan) in Patients with Relapsed Small Cell Lung Cancer (Sclc; Lagoon Trial). J. Clin. Oncol..

[16] Machiels J.-P., Staddon A., Herremans C., Keung C., Bernard A., Phelps C., Khokhar N.Z., Knoblauch R., Parekh T.V., Dirix L. (2014). Impact of cytochrome P450 3A4 inducer and inhibitor on the pharmacokinetics of trabectedin in patients with advanced malignancies: Open-label, multicenter studies. Cancer Chemother. Pharmacol..

[17] Iversen D.B., Andersen N.E., Dunvald A.D., Pottegård A., Stage T.B. (2022). Drug metabolism and drug transport of the 100 most prescribed oral drugs. Basic Clin. Pharmacol. Toxicol..

[18] Clozel M., Breu V., A Gray G., Kalina B., Löffler B.M., Burri K., Cassal J.M., Hirth G., Müller M., Neidhart W. (1994). Pharmacological Characterization of Bosentan, a New Potent Orally Active Nonpeptide Endothelin Receptor Antagonist. J. Pharmacol. Exp. Ther..

[19] Weber C., Gasser R., Hopfgartner G. (1999). Absorption, Excretion, and Metabolism of the Endothelin Receptor Antagonist Bosentan in Healthy Male Subjects. Drug Metab. Dispos..

[20] Aviles P., Altares R., van Andel L., Lubomirov R., Fudio S., Rosing H., del Pino F.M.M., Tibben M.M., Benedit G., Nan-Offeringa L. (2022). Metabolic Disposition of Lurbinectedin, a Potent Selective Inhibitor of Active Transcription of Protein-Coding Genes, in Nonclinical Species and Patients. Drug Metab. Dispos..

[21] Leary A., Oaknin A., Trigo J.M., Moreno V., Delord J.-P., Boni V., Braña I., Fernández C., Kahatt C., Nieto A. (2023). Pooled Safety Analysis of Single-Agent Lurbinectedin in Patients With Advanced Solid Tumours. Eur. J. Cancer.

[22] Elez M.E., Tabernero J., Geary D., Macarulla T., Kang S.P., Kahatt C., Pita A.S.-M., Teruel C.F., Siguero M., Cullell-Young M. (2014). First-In-Human Phase I Study of Lurbinectedin (PM01183) in Patients with Advanced Solid Tumors. Clin. Cancer Res..

[23] Gaillard S., Oaknin A., Ray-Coquard I., Vergote I., Scambia G., Colombo N., Fernandez C., Alfaro V., Kahatt C., Nieto A. (2021). Lurbinectedin Versus Pegylated Liposomal Doxorubicin or Topotecan in Patients with Platinum-Resistant Ovarian Cancer: A Multicenter, Randomized, Controlled, Open-Label Phase 3 Study (Corail). Gynecol. Oncol..

[24] King N., Garcia-Martinez S., Alcaraz E., Grisalena A., Lubomirov R., Altares R., Fernandez-Teruel C., Francesch A.M., Aviles P.M., Fudio S. (2023). Quantitative Determination of Lurbinectedin, Its Unbound Fraction and Its Metabolites in Human Plasma Utilizing Ultra-Performance Lc-Ms/Ms. PLoS ONE.

