# Supplementary material for: Impact of a Moderate CYP3A4 Inducer (Bosentan) on Lurbinectedin Pharmacokinetics and Safety in Patients with Advanced Solid Tumors: An Open-Label, Two-Way, Crossover, Phase Ib Drug–Drug Interaction Study
Source: Pharmaceuticals (Basel). 2024 Jan 30;17(2):182. doi: 10.3390/ph17020182 (PMC10892269; doi:10.3390/ph17020182)
Supplement: Supplementary file 1 [file pharmaceuticals-17-00182-s001.zip › pharmaceuticals-2777934-supplementary.pdf]

Impact of a Moderate CYP3A4 Inducer (Bosentan) on Lurbinectedin Pharmacokinetics and Safety in Patients with Advanced Solid Tumors: An Open-Label, Two-Way, Crossover, Phase Ib Drug–Drug Interaction Study

Irene Moreno, Tatiana Hernández, Emiliano Calvo, Salvador Fudio, Carmen Kahatt, Cristian Fernández, Jorge Luis Iglesias, Gema Corral, Laura Pérez-Ramos, Lola Montilla, Ali Zeaiter and Rubin Lubomirov.

<u>Supplementary material</u>

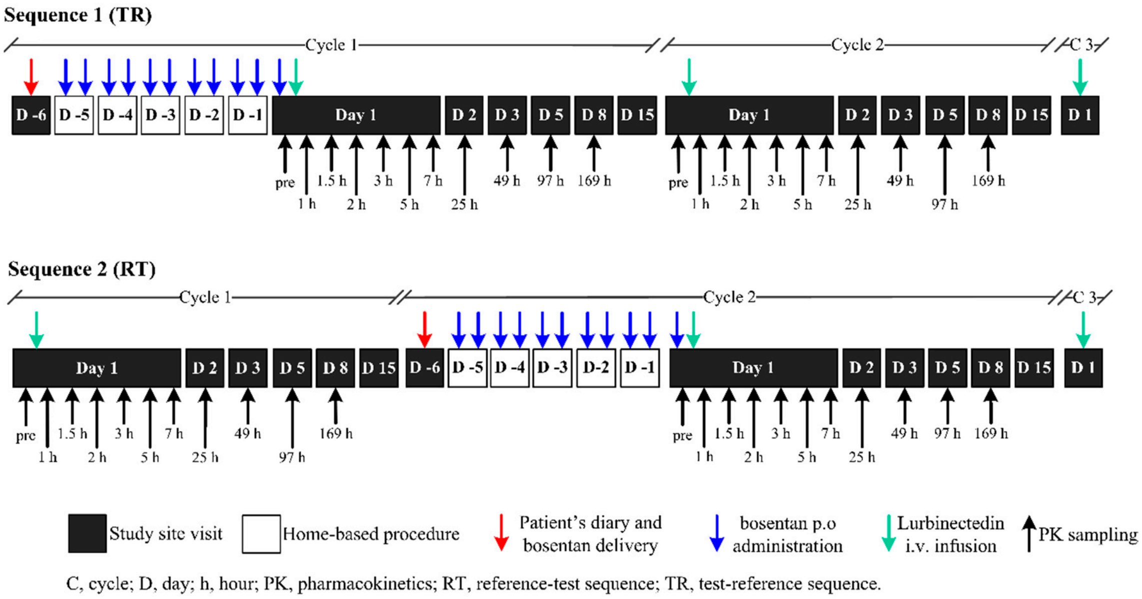

Figure S1. Schematic diagram of trial design. Note: in Sequence 2 (RT) the Day 15 (D15) of Cycle 1 and the Day –6 (D–6) of Cycle 2 represent the same day/visit in the absence of delays. Patients were randomized to receive either Sequence 1 (TR) of bosentan with lurbinectedin in Cycle 1 followed by two consecutive cycles of lurbinectedin alone [last cycle being optional]) or Sequence 2 (RT) of lurbinectedin alone in Cycle 1, bosentan with lurbinectedin in Cycle 2 and lurbinectedin alone in Cycle 3 (optional).
